# Overground running incurs a higher energetic cost than treadmill running at a 1% grade: A comparison of running economy, oxygen cost of transport, and energy cost in endurance athletes

**DOI:** 10.1371/journal.pone.0355988

**Published:** 2026-08-17

**Authors:** Seyed Houtan Shahidi, Rana Can, Furkan Murat Paça, Muhammed Doğukan Zengin

**Affiliations:** Faculty of Sports Sciences, Department of Sports Coaching, Istanbul Gedik University, Istanbul, Turkey; University degli Studi di Milano, ITALY

## Abstract

This study compared the physiological and metabolic demands of treadmill and overground running by examining running economy (RE), oxygen cost of transport (O2-COT), and energy cost (EC) across ventilatory threshold-scaled intensities in trained endurance athletes. Twelve male runners (21.3‍±‍2.4 years; 176.2‍±‍6.8 cm; 67.8‍±‍5.9 kg) completed a ramp test to determine V̇O2max, followed by identical stepwise protocols (8–15 km·h‍1‍¹; 3-min stages) performed indoors on a treadmill and outdoors on a 400 m track. Breath-by-breath gas exchange was averaged over the final 60–90 s of each stage to determine steady-state V̇O2. Within-subject comparisons were used to evaluate differences between field and treadmill running across exercise intensities. Two-way repeated-measures ANOVA revealed significant main effects of Condition and Intensity domain for all three primary outcomes (all p‍≤‍0.012). RE was significantly higher during overground running (51.3‍±‍2.2 vs. 49.6‍±‍3.1 mL·kg‍1‍¹·min‍1‍¹; p-FDR‍=‍0.01), with divergence occurring primarily at intensities between 70% and 100% of VT2. O2-COT was consistently greater during overground running across all intensity domains (204.2‍±‍11.3 vs. 197.1‍±‍12.3 mL·kg‍1‍¹·km‍1‍¹; p-FDR‍≤‍0.01), with no significant Condition‍×‍Intensity interaction, indicating a consistent between-condition difference across all intensity domains. Energy cost remained consistently higher during overground running (3.87‍±‍0.21 to 4.60‍±‍0.27 vs. 3.65‍±‍0.22 to 4.10‍±‍0.18 J·kg‍1‍¹·m‍1‍¹; p-FDR‍<‍0.001), with a significant Condition‍×‍Intensity interaction (p‍=‍0.045). Carbohydrate oxidation was significantly higher during overground running at intensities approaching VT2 (p-FDR‍=‍0.02). These findings suggest that the commonly used 1% treadmill grade correction does not fully replicate the energetic demands of overground running, with implications for laboratory-based performance evaluation.

## Introduction

Endurance running imposes sustained demands on the cardiovascular, respiratory, and muscular systems, requiring a finely tuned balance between energy supply and utilization across varying exercise intensities [1]. Running economy (RE) reflects the steady-state oxygen cost of maintaining a specific running speed. It is typically expressed relative to body mass (mL·kg‍1‍¹·min‍1‍¹) because the absolute metabolic requirement is proportional to an individual’s body mass and morphology, allowing meaningful comparison of the oxygen cost between runners at a given speed [2]. The oxygen cost of transport (O2-COT) quantifies this cost per unit distance (mL·kg‍1‍¹·km‍1‍¹), facilitating comparisons across running speeds [3,4]. Measurements of substrate oxidation patterns provide important additional metabolic information for interpreting both EC and O2-COT [5]. The energy cost of transport (EC), expressed in joules or kilocalories per unit distance (J·kg‍1‍¹·m‍1‍¹), integrates the caloric equivalent of oxygen based on substrate oxidation rates [6–10].

Exercise intensity is commonly categorized into physiological domains defined by ventilatory thresholds. The first ventilatory threshold (VT₁) marks the transition from moderate to heavy exercise, when ventilation increases disproportionately relative to oxygen uptake. The second ventilatory threshold (VT2) demarcates the heavy-to-severe intensity boundary, beyond which metabolic demands rise steeply, and fatigue accumulates rapidly. Anchoring comparisons between running conditions to these individually determined thresholds is particularly relevant when evaluating treadmill versus overground running, as fixed absolute speeds may place individuals at different relative intensities depending on the environment. Threshold-based comparisons therefore provide a more physiologically meaningful framework for detecting condition-specific differences in energetic and metabolic responses.

Elite endurance runners typically demonstrate O2-COT values of approximately 180–200 mL·kg‍1‍¹·km‍1‍¹ at a velocity of 16 km·h‍1‍¹ [11]. The gold standard for quantifying cardiorespiratory and metabolic responses during running remains laboratory-based cardiopulmonary exercise testing (CPET), typically performed using incremental or stepwise treadmill protocols [12]. To improve ecological validity, a 1% treadmill gradient has historically been recommended to match overground running energetics [13]. However, a recent meta-analysis by Miller et al. (2019), synthesizing 53 crossover studies involving a mixed population of trained endurance athletes, recreationally active individuals, and sedentary participants, found that treadmill and overground running elicit broadly comparable physiological responses at submaximal speeds up to approximately 16 km·h‍1‍¹, calling into question the universal applicability of this adjustment [14]. Beyond physiological considerations, treadmill and overground running also produce comparable mechanical responses at many speeds. Specifically, the 1% rule may substantially overestimate or underestimate the contribution of air drag to the mechanical work of running, depending on running speed [15].

Collectively, these findings suggest that it remains unclear whether the 1% treadmill grade correction is a valid physiological principle [16]. One possible explanation for the conflicting evidence may be differences in how energy demand has been expressed across studies [17]. While previous studies have examined the metabolic comparability of treadmill and overground running using time-normalized oxygen uptake at broadly defined submaximal speeds, they have not incorporated distance-normalized outcomes such as O2-COT and EC, nor anchored comparisons to individually determined physiological thresholds, nor examined substrate utilization alongside energetic cost [13,14].

Therefore, the present study aimed to compare RE, O2-COT, and EC between overground running and treadmill running performed at a 1% grade in trained endurance athletes, using a standardized within-subject protocol. Integrative assessments incorporating RE, O2-COT, and EC may provide a more sensitive framework for detecting differences between overground and 1%-grade treadmill running than oxygen uptake alone. We hypothesized that, at matched submaximal speeds, overground running would be associated with higher RE, O2-COT, and EC compared with 1%-grade treadmill running, particularly at intensities approaching VT2. We further hypothesized that overground running would elicit a greater reliance on carbohydrate oxidation relative to fat oxidation, specifically at intensities at and above VT₁, where the additional mechanical and neuromuscular demands of overground running would be expected to increase glycolytic flux and elevate the respiratory exchange ratio.

## Methods

### Ethics statement

The study protocol was approved by the University Health Sciences Ethics Committee (Approval No: E-11470191-050.04-2025.173340.33; Date: 02/06/2025) and conducted in accordance with the Declaration of Helsinki. Participant recruitment and data collection were carried out between 01/07/2025 and 01/08/2025. All participants received verbal and written explanations of the study purpose, procedures, and potential risks before providing written informed consent.

### Participants

Twelve endurance-trained male athletes voluntarily participated in this study. Their mean age was 21.3‍±‍2.4 years, height 176.2‍±‍6.8 cm, body mass 67.8‍±‍5.9 kg, and body fat percentage 11.8‍±‍3.2%. All participants were nationally licensed runners training‍≥‍5 sessions per week, corresponding to a weekly running volume of approximately 65–80 km. Based on the Participant Classification Framework [18], these athletes were classified as Tier 3: Highly Trained/National-Level. All of the athletes were free from cardiovascular, pulmonary, metabolic, or musculoskeletal disorders. Exclusion criteria included smoking, chronic illness, and the use of any medication affecting cardiorespiratory function. Only male participants were recruited to minimize the confounding influence of menstrual cycle–related hormonal fluctuations on ventilatory and metabolic responses, ensuring greater homogeneity in physiological comparisons.

### Study design

**Fig 1:**
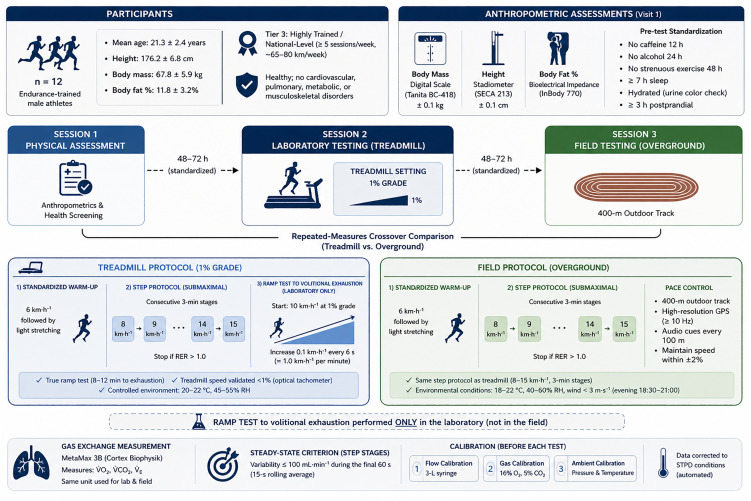
Study Design Schematic.

This study used a repeated-measures crossover design to compare overground running with treadmill running performed at a 1% grade. An a priori power analysis was conducted using G*Power version 3.1 (Heinrich-Heine-Universität Düsseldorf, Germany) for a paired-samples t-test, assuming a large within-subject effect size (Cohen’s dz‍=‍0.8), an alpha level of 0.05, and a statistical power of 0.80 [19]. The selected effect size was grounded in the existing literature on running economy differences between treadmill and overground conditions. Specifically, Morgan et al. (1994) reported mean RE differences of approximately 2–3 mL·kg‍1‍¹·min‍1‍¹ between treadmill and overground running in trained male runners [20], while Saunders et al. (2004) identified a 2–3% change in RE as the minimum physiologically meaningful difference in trained endurance athletes [5]. Assuming a representative submaximal V̇O2 of approximately 50 mL·kg‍1‍¹·min‍1‍¹, a 4% difference corresponds to a mean difference of ~2 mL·kg‍1‍¹·min‍1‍¹. Drawing on within-subject standard deviations of repeated RE measurements reported in comparable crossover designs (CV approximately 2.5–3%; Morgan et al., 1994), this yields an SD of differences of approximately 1.5–2.5 mL·kg‍1‍¹·min‍1‍¹, and a resulting Cohen’s dz of approximately 0.80–1.30. A conservative value of dz‍=‍0.80 was therefore selected as the basis for the power calculation. This analysis indicated that a minimum sample size of 12 participants was required. The study consisted of three testing sessions: an initial physical assessment, a treadmill-based session (Fig 1), and a field-based assessment. The order of testing was not randomized; all participants completed the treadmill-based session prior to the field-based assessment. The time interval between testing sessions was standardized across participants, with a minimum of 48 hours and a maximum of 72 hours between visits to minimize residual fatigue and training effects.

### Anthropometric assessments

During the first laboratory visit, participants underwent standardized anthropometric assessments. Body mass was measured to the nearest 0.1 kg using a calibrated digital scale (Tanita BC-418, Tanita Corporation, Tokyo, Japan), and standing height was measured to the nearest 0.1 cm using a stadiometer (SECA 213, SECA GmbH, Hamburg, Germany). Body fat percentage was estimated via multi-frequency bioelectrical impedance analysis (InBody 770, InBody Co. Ltd., Seoul, South Korea). Before each testing session, participants were instructed to refrain from caffeine for 12 hours, alcohol for 24 hours, and strenuous exercise for 48 hours prior to testing. To minimize further physiological variability, participants arrived well-rested (verified via verbal confirmation of at least 7 hours of sleep), adequately hydrated (confirmed by visual inspection of urine color using a standard urine color chart; no urine samples were collected), and at least three hours postprandial.

### Laboratory testing

The treadmill protocol was conducted on a motorized treadmill (TrackMaster TMX425C, Full Vision Inc., Newton, KS, USA) connected to the CORTEX Metamax software via an RS-232 cable, which enabled automatic control of treadmill speed throughout all testing sessions. Data were collected under the commonly employed 1% treadmill grade, which is conventionally used to match the oxygen cost of overground running at a given speed by offsetting the absence of air resistance in laboratory settings [13].

**Fig 2:**
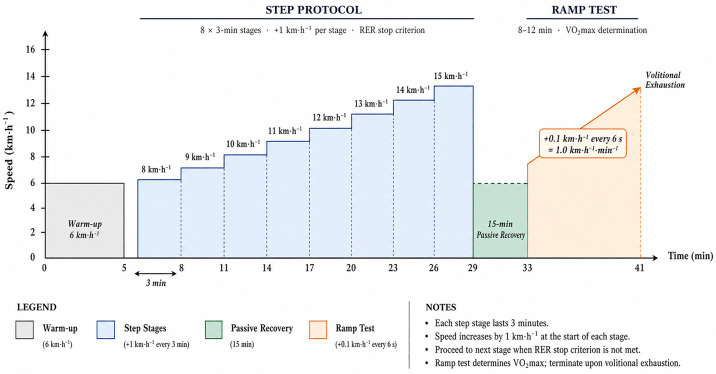
Protocol Design Schematic. Note. Participants completed a standardized treadmill protocol beginning with a 5-minute warm-up at 6 km·h‍1‍¹, followed by 3-minute incremental stages at 8–15 km·h‍1‍¹ (step protocol). After completion of the step test, a recovery phase was performed, followed by a continuous ramp protocol starting at submaximal intensity and progressing to volitional exhaustion.

An identical standardized warm-up (6 km·h‍1‍¹ followed by light stretching) was performed before testing. The step protocol then commenced, consisting of consecutive three-minute stages beginning at 8 km·h‍1‍¹ and increasing automatically by 1 km·h‍1‍¹ per stage up to 15 km·h‍1‍¹, with testing discontinued when the respiratory exchange ratio exceeded 1.0. After a 15-minute seated recovery, participants performed a continuous ramp test beginning at 10 km·h‍1‍¹ at a 1% gradient until volitional exhaustion. Treadmill speed was increased automatically by 0.1 km·h‍1‍¹ every 6 seconds, equivalent to a rate of 1.0 km·h‍1‍¹ per minute, producing a true ramp rather than a step incremental protocol (As shown in Fig 2). This design was selected to induce maximal fatigue within approximately 8–12 minutes, consistent with established recommendations for ramp test duration. Treadmill belt speed was validated before each session using a handheld optical tachometer (Shimpo DT-2100, Japan) to ensure less than 1% deviation from the display speed [15].

### Field testing

The field test replicated the treadmill step protocol and was conducted on a 400-m outdoor track. After a standardized warm-up at 6 km·h‍1‍¹ followed by light stretching, athletes completed consecutive three-minute stages at speeds ranging from 8 to 15 km·h‍1‍¹. Running speed was continuously monitored using a high-resolution GPS device with a minimum sampling frequency of 10 Hz (Garmin, USA), while audio cues provided every 100 meters helped participants maintain their target pace within a 2% margin of error. This tolerance was based on the positional accuracy of the GPS device used (Garmin, 10 Hz; manufacturer-reported accuracy ±2–3 m), which corresponds to a pace deviation of approximately 1–2% at the speeds tested (8–13 km·h‍1‍¹), combined with the practical constraint that participants received corrective audio feedback every 100 meters to adjust their speed accordingly. The ramp test to volitional exhaustion was performed exclusively in the laboratory following completion of the step protocol and was not conducted during field testing.

### Gas Exchange measurement and calibration procedures

Gas exchange variables (oxygen uptake, V̇O2; carbon dioxide output, V̇CO2; and minute ventilation, V̇E) were assessed using a portable metabolic analyzer (MetaMax 3B, Cortex Biophysik GmbH, Leipzig, Germany), which exhibits excellent test–retest reliability for V̇O2 (coefficient of variation <2%) and strong validity compared with Douglas bag reference systems (mean bias <2%; r‍>‍0.98) [21]. Before each test, the system underwent a standardized three-step calibration procedure: (1) flowmeter calibration using a 3-L syringe (Hans Rudolph Inc., Kansas City, MO, USA); (2) gas calibration with certified reference gases (16% O2, 5% CO2; Air Liquide, France); and (3) ambient pressure and temperature calibration [22]. Steady-state V̇O2 was confirmed when variability was‍≤‍100 mL·min‍1‍¹ during the final 60 seconds of each 3-minute stage, using a 15-second rolling average to minimize breath-by-breath fluctuations, criteria consistent with established recommendations for submaximal steady-state verification [7]. The same MetaMax 3B unit was used for both laboratory and field assessments to ensure methodological consistency. Laboratory tests were conducted under controlled environmental conditions (20–22 °C, relative humidity 45–55%). Field trials were performed during late afternoon/early evening (18:30–21:00), when ambient temperatures were within a mild, thermoneutral range (18–22 °C; relative humidity 40–60%; wind speed <3 m·s‍1‍¹). Ambient temperature, pressure, and humidity were recorded before each test session and entered into the MetaMax 3B system before data collection, allowing automated correction of gas volumes to standard temperature and pressure dry (STPD) conditions in accordance with the manufacturer’s calibration protocol.

### Maximal oxygen uptake

V̇O2max was determined as the highest 30-second mean V̇O2 obtained during the ramp test. Attainment of V̇O2max was verified when at least two of the following criteria were met: (a) a plateau in V̇O2 despite increased workload (change of less than 150 mL·min‍1‍¹), (b) respiratory exchange ratio (RER) ≥ 1.10, (c) heart rate‍≥‍95% of age-predicted maximum, and (d) rating of perceived exertion (RPE) ≥ 19 on the Borg 6–20 scale [23]. If fewer than two criteria were satisfied, the test was not accepted as a true maximal effort. In such cases, participants were given an additional 30-minute passive recovery period and asked to repeat the ramp test.

### Ventilatory threshold determination

Ventilatory thresholds were determined according to the classical framework described by Wasserman and colleagues using a multi-criteria approach. The first ventilatory threshold (VT₁) was identified using the V-slope method, defined as the breakpoint at which CO2 increased disproportionately relative to V̇O2, indicating the onset of excess CO2 production [24]. This determination was corroborated by a systematic increase in V̇E/V̇O2 without a concomitant increase in V̇E/V̇CO2, alongside a rise in end-tidal oxygen pressure (PetO2) with stable end-tidal carbon dioxide pressure (PetCO2) [25,26]. The second ventilatory threshold (VT2) was identified as the exercise intensity at which V̇E/V̇CO2 increased systematically, reflecting the onset of respiratory compensation for metabolic acidosis. A decline in PetCO2 further confirmed this point, a continued rise in PetO2, and a disproportionate increase in minute ventilation relative to both V̇O2 and V̇CO2. Ventilatory equivalents and end-tidal gas variables were used exclusively for ventilatory threshold determination and are therefore not presented as standalone outcome variables. Threshold identification was performed independently by two experienced exercise physiologists. Agreement between raters was assessed using intraclass correlation coefficients (ICC, two-way mixed model, absolute agreement). ICC values exceeded 0.90 for both VT₁ and VT2 across all participants, indicating excellent inter-rater reliability. In cases where the two estimates differed by more than one stage, the discrepancy was resolved through consensus discussion between the two physiologists. Where estimates were within one stage of each other, the mean of the two values was used as the final threshold.

### Running economy

RE was determined as the mean relative oxygen consumption (mL·kg‍1‍¹·min‍1‍¹) averaged over the final 60–90 seconds of each three-minute stage, representing the steady-state V̇O2 for that intensity.[5].

Absolute oxygen uptake in liters per minute (L·min‍1‍¹) was also reported for further comparisons [27]. Oxygen Cost of Transport. The O2-COT was calculated as the oxygen cost required to cover one kilometer using the following equation: ${\text{O}}_{\text{2}}-\text{COT}=\frac{\text{V}{\text{O}}_{\text{2}}\;(\text{mL}\cdot \text{kg}-\text{1}\cdot \text{min}-\text{1})}{\text{speed}\;(\text{m}\cdot \text{min}-\text{1})}$ × 1000. Where V̇O2 represents the steady-state oxygen consumption for each three-minute stage.

### Energy cost

To integrate substrate oxidation into the overall energetic demand, EC of running was calculated in accordance with Fletcher et al. (200D) [7]. The energetic equivalent of oxygen (EEO2) was derived from the respiratory exchange ratio (RER) using the continuous equation of Peronnet and Massicotte (1991): EEO2 (kJ·L‍1‍¹ O2) = 4.585‍×‍RER‍+‍16.58. For computational consistency, EEO2 values were converted to joules per millilitre of oxygen (J·mL‍1‍¹ O2) before calculation [9]. Energy cost was then calculated as: EC (J·kg‍1‍¹·m‍1‍¹) = (V̇O2 [mL·kg‍1‍¹·min‍1‍¹] × EEO2 [J·mL‍1‍¹ O2]) / speed [m·min‍1‍¹]. Energy cost values are primarily reported in SI units (J·kg‍1‍¹·m‍1‍¹). For interpretability and comparison with previous literature, EC values were additionally expressed as kcal·kg‍1‍¹·km‍1‍¹ using standard unit conversions (1 kcal‍=‍4184 J). An equation-based approach was preferred over stoichiometric tables to enable continuous estimation of energy equivalents from breath-by-breath RER data during incremental exercise.

### Substrate utilization

Substrate oxidation rates for fat and carbohydrate were determined from the simultaneous measurement of V̇O2 and V̇CO2 using standard non-protein respiratory stoichiometric equations [28]. Protein oxidation was assumed to be negligible, as the exercise durations were short and participants were in a post-absorptive state. The equations applied were as follows: Fat oxidation (g·min‍1‍¹) = 1.695‍×‍V̇O2 - 1.701‍×‍V̇CO2. Carbohydrate oxidation (g·min‍1‍¹) = 4.210‍×‍V̇CO2 - 2.962‍×‍V̇O2, where both V̇O2 and V̇CO2 were expressed in liters per minute [28]. Substrate utilization, RE, O2-COT, and EC were analyzed only for stages in which the respiratory exchange ratio (RER) was‍≤‍1.00, ensuring steady-state metabolic conditions and minimizing the confounding effects of excess CO2 from bicarbonate buffering. Stages exceeding this threshold were excluded from substrate and energetic analyses but were retained for ventilatory variables (specifically V̇E, V̇E/V̇O2, V̇E/V̇CO2, PetO2, and PetCO2, which are used exclusively for threshold determination) where appropriate. Note that RPE was recorded at the end of each stage, but perceptual data are not reported as a primary outcome of this study, as the focus was on energetic and metabolic comparisons between conditions. This approach aligns with methodological recommendations by Fletcher et al. (200D) [7]. The potential influence of non-metabolic CO2 above VT₁ on RER-based substrate estimates, and the possible emergence of a V̇O2 slow component at higher submaximal intensities, are acknowledged as methodological considerations and are discussed further in the Study Limitations section.

### Steady-state verification

Steady-state conditions for V̇O2 and V̇CO2 were verified before calculating RE and O2-COT. A steady state was confirmed when [1] the linear slope of V̇O2 across the final 60 s of each 3-min stage was‍≤‍100 mL·min‍1‍¹·min‍1‍¹ and [2] the coefficient of variation (CV) for both V̇O2 and V̇CO2 during that period was‍≤‍5%. If either criterion was not met, the data from that stage were excluded from RE and O2-COT analysis. Consistent with established recommendations only running speeds at or below the individual’s VT2 were included in the primary RE and O2-COT analysis to ensure steady-state validity [7].

### Intensity domain segmentation

To facilitate physiologically meaningful comparisons, responses were segmented into three intensity domains defined as percentages of VT2 speed (70–79%, 80–89%, and 90–100% of VT2), anchored to treadmill-derived VT₁ and VT2 speeds as a common reference for both conditions. This approach was deemed appropriate given the homogeneity of the sample, as all participants were nationally licensed endurance athletes of comparable training status, with VT₁ and VT2 speeds remaining consistent across individuals and comparable between conditions. The selected intensity bands therefore correspond to the heavy-intensity domain spanning the VT₁–VT2 interval for all participants, where condition-specific differences in energetic cost are most physiologically relevant [29,30].

### Statistical analysis

Descriptive results are reported as mean‍±‍standard deviation (SD) with 95% confidence intervals (95% CI). Data normality was confirmed with the Shapiro–Wilk test. For between-condition comparisons at each matched velocity/intensity, paired-samples t-tests were used. When multiple comparisons were performed across speeds or VT2-related intensity bands, *p*-values were adjusted using the Benjamini–Hochberg false discovery rate (FDR) procedure [31]. Effect sizes were expressed as Cohen’s dz (mean difference / SD of differences), interpreted as small (0.20), medium (0.50), and large (0.80) [32] to correct for small-sample bias; Hedges’ gₐ was also reported. For the primary outcomes (RE, O2-COT, EC), a two-way repeated-measures ANOVA was conducted with Condition (lab vs. field) and Intensity domain (70–79%, 80–89%, 90–100% VT2) as within-subject factors. Where sphericity was violated, Greenhouse-Geisser corrected p-values are reported. Effect sizes are reported as generalised eta-squared (ηg²). Regression equations (intercept, slope), Pearson’s correlation coefficient (r), coefficient of determination (R²), and adjusted R² are reported for each condition; these analyses were considered secondary and primarily descriptive. All analyses were conducted using OriginPro 2024 (OriginLab, Northampton, MA, USA), with statistical significance accepted at α‍=‍0.05.

## Results

### Participant characteristics

All participants completed both laboratory and field tests without adverse events. Participant characteristics and key physiological outcomes are summarized in Table 1. Briefly, absolute and relative V̇O2max did not differ significantly between conditions (p‍=‍0.23 and p‍=‍0.28, respectively). VT₁ speed was also comparable between conditions (p‍=‍0.16). Notably, VT2 occurred at a significantly higher running speed during overground running compared with treadmill running (p‍=‍0.02, dz‍=‍0.68).

**Table 1:** Physiological and metabolic responses during laboratory and field-based incremental running tests.

Parameter	Lab (Mean ± SD)	Field (Mean ± SD)	Δ [95% CI]	p-value	Effect size (dz / ηp²)
**Maximal capacity**					
**Absolute V̇O2max (L·min1¹)**	4.22 ± 0.35	4.28 ± 0.38	+0.06 [−0.04, +0.16]	0.23	dz = 0.25
**Relative V̇O2max (mL·kg1¹·min1¹)**	62.4 ± 4.8	63.5 ± 5.2	+1.1 [−0.9, +3.0]	0.28	dz = 0.22
**Ventilatory thresholds-speed**					
**VT₁ (km·h1¹)**	10.3 ± 0.7	10.5 ± 0.6	+0.2 [−0.1, +0.5]	0.16	dz = 0.30
**VT2 (km·h1¹)**	13.1 ± 0.8	13.5 ± 0.7	+0.4 [+0.1, +0.7]	0.02*	dz = 0.68
**Ventilatory thresholds-relative intensity**					
**VT₁ (%V̇O2max)**	72.1 ± 4.9	73.4 ± 5.1	+1.3 [−0.9, +3.5]	0.24	dz = 0.25
**VT2 (%V̇O2max)**	88.6 ± 3.7	90.8 ± 4.0	+2.2 [+0.6, +3.8]	0.01*	dz = 0.72
**V̇O2 at ventilatory thresholds †**					
**V̇O2 at VT₁ (mL·kg1¹·min1¹)**	45.0 ± 4.6	46.6 ± 5.0	+1.6 [−0.5, +3.7]	0.13	dz = 0.43†
**V̇O2 at VT₁ (L·min1¹)**	3.05 ± 0.41	3.16 ± 0.44	+0.11 [−0.08, +0.30]	0.24	dz = 0.33†
**V̇O2 at VT2 (mL·kg1¹·min1¹)**	55.3 ± 4.8	57.7 ± 5.4	+2.4 [+0.1, +4.7]	0.04*	dz = 0.60†
**V̇O2 at VT2 (L·min1¹)**	3.75 ± 0.46	3.91 ± 0.50	+0.16 [−0.05, +0.37]	0.13	dz = 0.43†
**Performance outcomes at 12 km·h1¹**					
**RE 12 km·h1¹ (mL·kg1¹·min1¹)**	46.9 ± 3.1	48.6 ± 2.9	+1.7 [0.5, 2.9]	0.01*	dz = 0.61
**O2-COT 12 km·h1¹ (mL·kg1¹·km1¹)**	197.1 ± 12.3	204.2 ± 11.8	+7.1 [2.4, 11.8]	0.01*	dz = 0.59
**EC 12 km·h1¹ (kcal·kg1¹·km1¹)**	0.87 ± 0.05	0.93 ± 0.05	+0.06 [−0.01, +0.44]	0.01*	dz = 0.60

Note. Values are mean‍±‍SD. Δ‍=‍mean difference (field – laboratory) with 95% CI. p-values are FDR-adjusted (p_FDR) for multiple comparisons. * p‍<‍0.05. Effect sizes: Cohen’s dz for paired contrasts; ηp² for repeated-measures effects. Abbreviations: VT‍=‍ventilatory threshold; V̇O2max‍=‍maximal oxygen uptake; RE‍=‍running economy; O2-COT‍=‍oxygen cost of transport; EC‍=‍energy cost.

### Running economy

**Fig 3:**
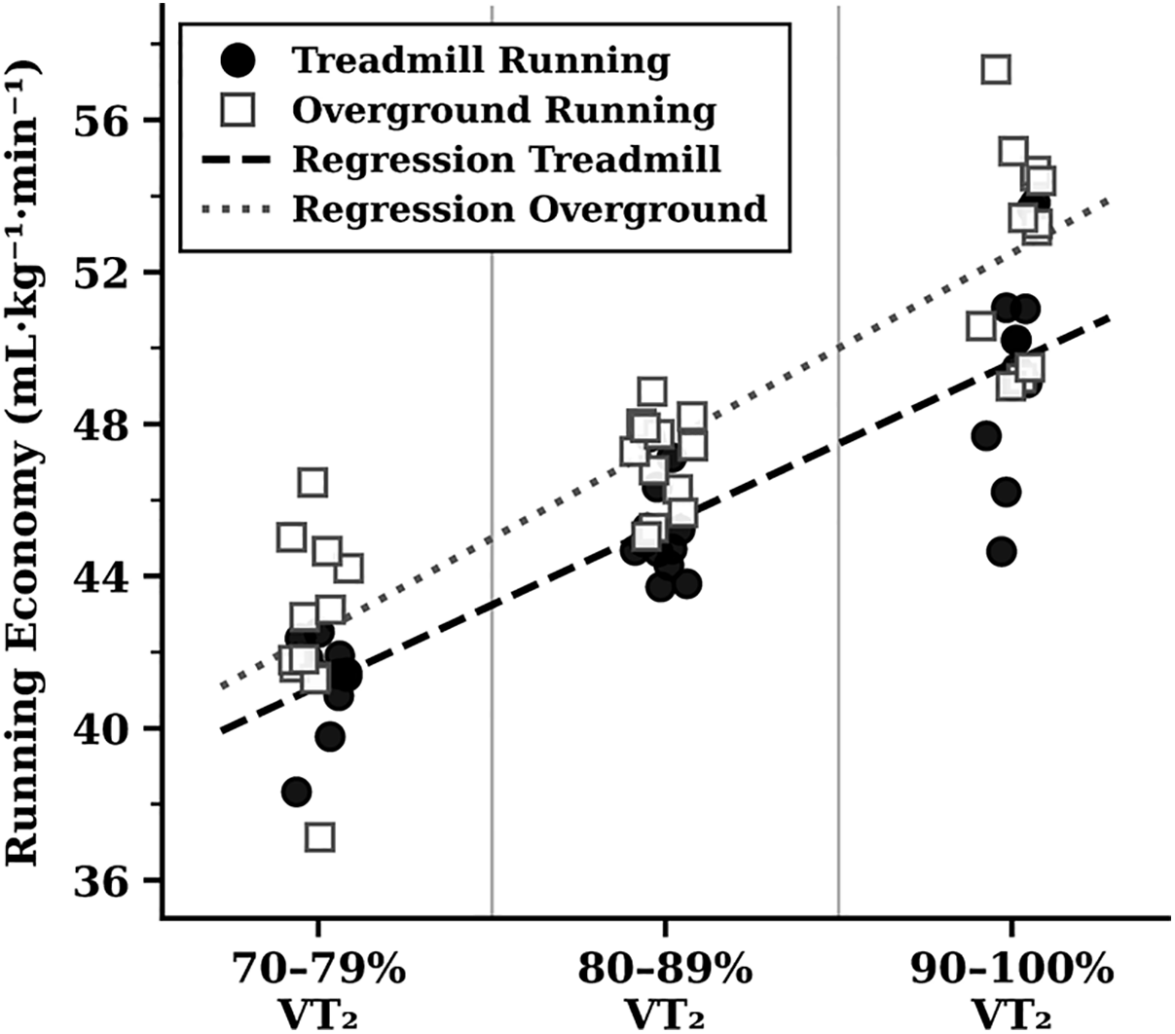
Comparison of running economy during treadmill and overground running. Note: Comparison of running economy (RE) across laboratory (treadmill) and overground running at intensities scaled relative to each athlete’s ventilatory threshold 2 (VT2). Each point represents an individual RE measurement (mL·kg‍1‍¹·min‍1‍¹), with blue circles denoting treadmill running and orange circles denoting overground running. Linear regression lines for each condition are shown (blue‍=‍treadmill, orange‍=‍overground running), with regression outcomes displayed in the inset.

For between-condition comparisons at a single reference speed, 12 km·h‍1‍¹ was selected as the common anchor point. This speed was chosen because it was the highest speed at which all twelve participants achieved verified steady-state V̇O2 in both conditions, placing it consistently within the heavy- intensity domain (between VT₁ and VT2) for all participants. It therefore represents the highest ecologically valid common speed for meaningful within-subject comparisons across conditions. A two-way repeated-measures ANOVA revealed a significant main effect of Condition (F(1,11) = 8.944, p‍=‍0.012, ηg²‍=‍0.112) and a significant main effect of Intensity domain (F(2,22) = 150.508, p‍<‍0.001, ηg²‍=‍0.841). The Condition‍×‍Intensity interaction was not significant (F(2,22) = 2.237, p‍=‍0.131, ηg²‍=‍0.056), indicating that the between-condition difference in RE was consistent across intensity domains. At this intensity, RE was significantly higher during overground running compared with treadmill running (51.3‍±‍2.2 vs. 49.6‍±‍3.1 mL·kg‍1‍¹·min‍1‍¹; Δ‍=‍+1.7‍±‍1.9 mL·kg‍1‍¹·min‍1‍¹, *p* ₍FDR₎‍=‍0.01, dz‍=‍0.61). Across VT2-relative intensity domains (70–79%, 80–89%, and 90–100%), RE increased progressively in both environments, with overground running demonstrating greater variability while preserving the expected positive relationship between relative intensity and oxygen cost. Linear regression analyses revealed a steeper RE-intensity slope during overground running (b‍=‍0.4189‍±‍0.04 mL·kg‍1‍¹·min‍1‍¹ per %VT2) compared with treadmill running (b‍=‍0.3446‍±‍0.039 mL·kg‍1‍¹·min‍1‍¹ per %VT2), while intercept values were similar between conditions.Strong associations were observed between intensity and RE in both settings (treadmill r‍=‍0.8344, R²‍=‍0.696; field r‍=‍0.8526, R²‍=‍0.727), as shown in Fig 3.

### Oxygen Cost of Transport

**Fig 4:**
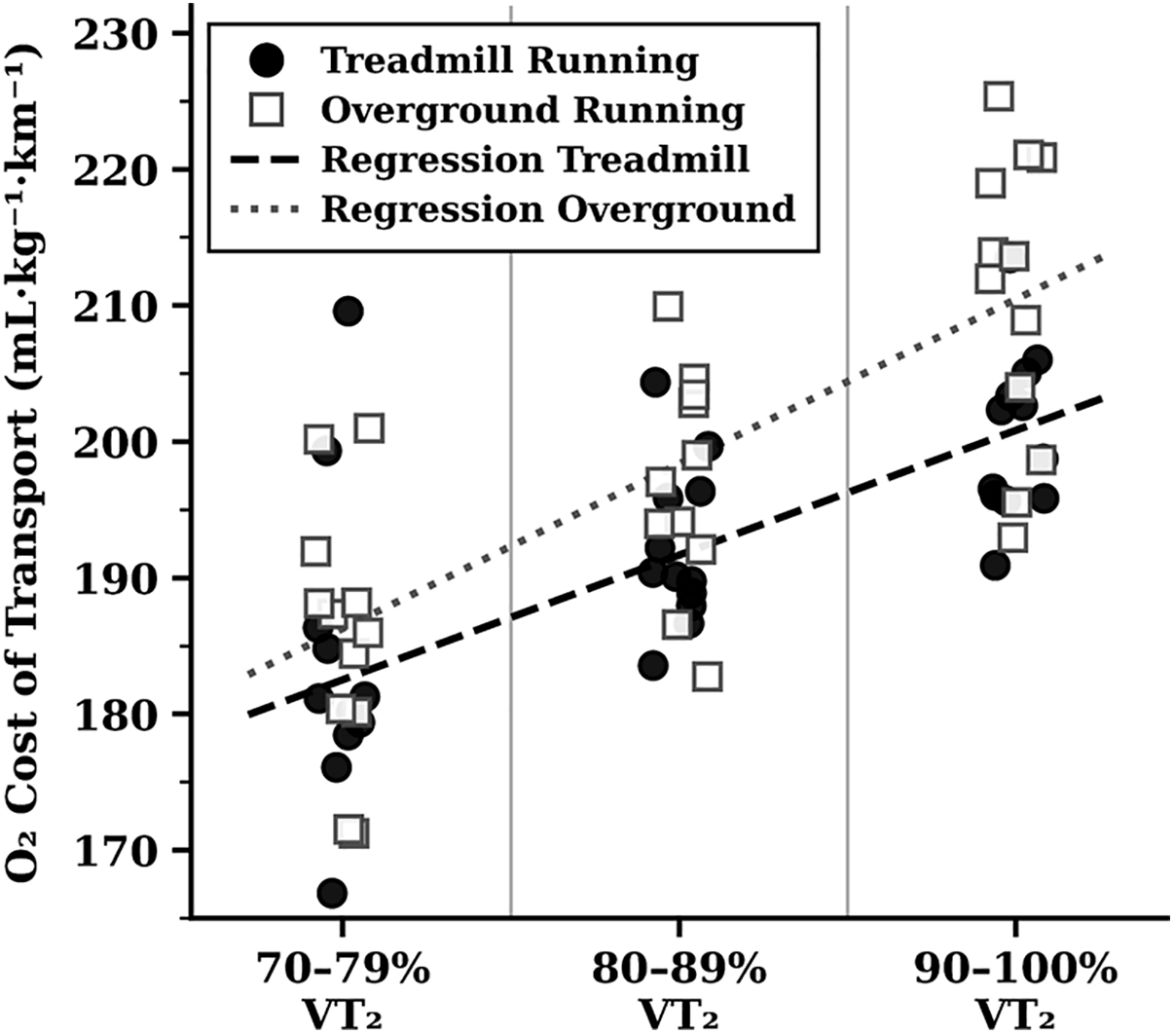
Comparison of the cost of transport during treadmill and overground running. Note: Oxygen cost of transport (O_2_-COT; mL·kg‍1‍¹·km‍1‍¹) during treadmill (blue) and overground running (orange) across three relative intensity domains normalized to ventilatory threshold 2 (VT2). Each point represents an individual measurement, with linear regression lines illustrating the relationship between increasing VT2-scaled intensity and metabolic cost. Regression parameters are shown in the inset.

The O2-COT was analyzed at the highest speed at which all participants achieved steady-state oxygen uptake between VT₁ and VT2. A two-way repeated-measures ANOVA revealed significant main effects of Condition (F(1,11) = 24.079, p‍<‍0.001, ηg²‍=‍0.192) and Intensity domain (F(2,22) = 65.781, p‍<‍0.001, ηg²‍=‍0.625). The Condition‍×‍Intensity interaction was not significant (F(2,22) = 0.702, p‍=‍0.506, ηg²‍=‍0.025), suggesting that overground O2-COT remained consistently higher than treadmill across all intensity domains. At this velocity, O2-COT was significantly higher during overground running compared with treadmill running (204.2‍±‍11.3 vs. 197.1‍±‍12.3 mL·kg‍1‍¹·km‍1‍¹), corresponding to a difference of +7.1‍±‍9.8 mL·kg‍1‍¹·km‍1‍¹ (*p* ₍FDR₎‍=‍0.01, dz‍=‍0.59). Across VT2-relative intensity domains (70–79% to 90–100%), both laboratory and field conditions demonstrated a clear positive relationship between metabolic load and COT. Field running exhibited a lower regression intercept (179.62‍±‍3.07 mL·kg‍1‍¹·km‍1‍¹) compared with treadmill running (182.81‍±‍2.86 mL·kg‍1‍¹·km‍1‍¹). The two regression lines converge at approximately 19% of VT2, well below the intensity domain examined, meaning overground O2-COT exceeded treadmill values across the entire 70–100% VT2 range shown in Fig 4, consistent with the steeper slope of the overground condition.

### Energy cost

**Fig 5:**
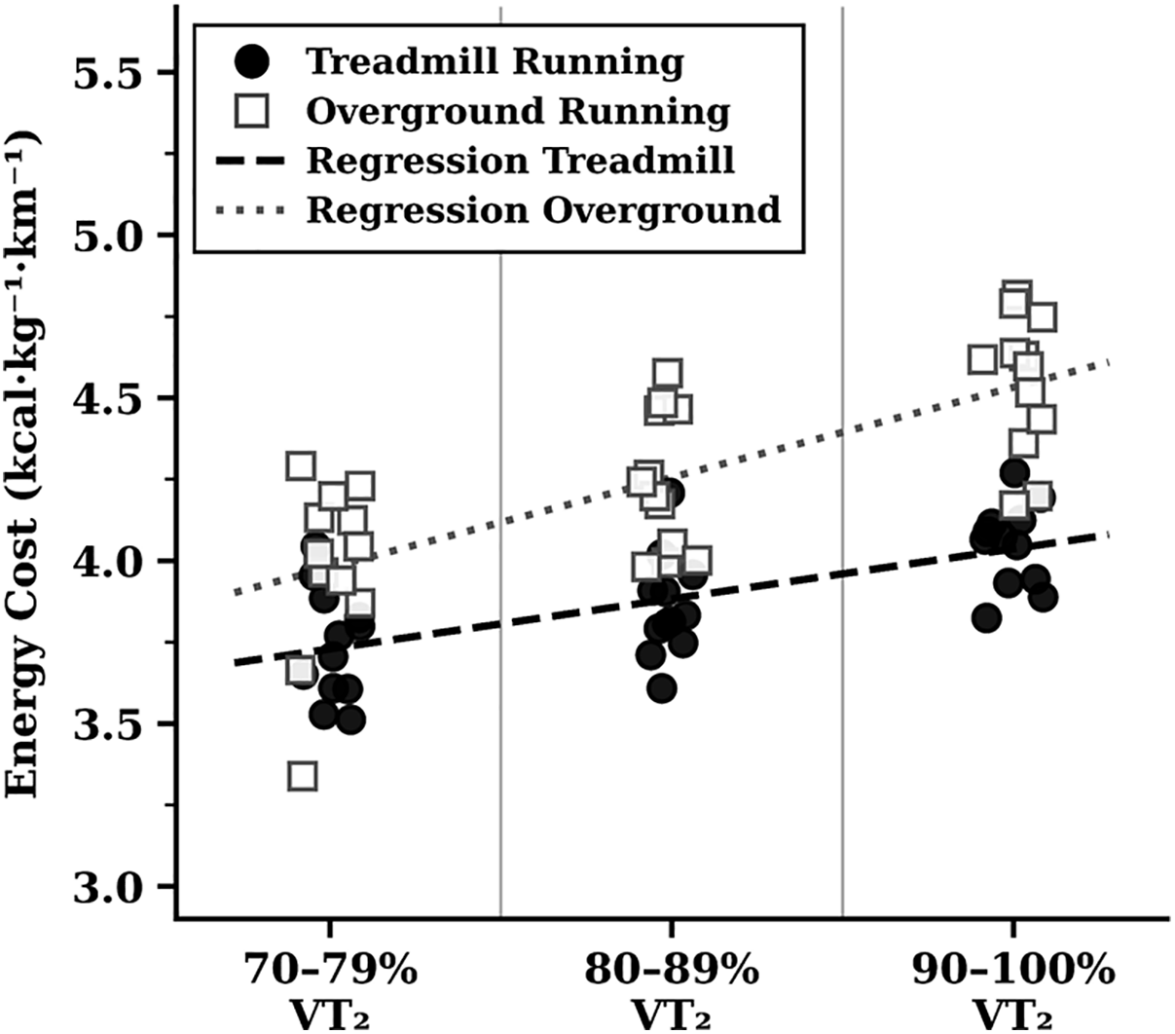
Comparison of Energy Cost during treadmill and overground running. Individual data points are shown with linear regression lines for each condition (blue‍=‍treadmill; orange‍=‍overground running), and regression statistics are provided in the inset. Note. Energy cost (EC; J·kg‍1‍¹·m‍1‍¹) during treadmill (blue) and overground running (orange) across relative intensity bands scaled to ventilatory threshold 2 (VT2). Individual data points are shown with linear regression lines for each condition (black‍=‍treadmill; orange‍=‍overground running), and regression statistics are provided in the inset.

A two-way repeated-measures ANOVA revealed significant main effects of Condition (F(1,11) = 30.330, p‍<‍0.001, ηg²‍=‍0.366) and Intensity domain (F(2,22) = 36.809, p‍<‍0.001, ηg²‍=‍0.542). A significant Condition‍×‍Intensity interaction was also observed (F(2,22) = 3.579, p‍=‍0.045, ηg²‍=‍0.075), indicating that the difference in EC between overground and treadmill running increased progressively with intensity. The EC at 12 km·h‍1‍¹ was significantly higher during overground running compared with treadmill running (3.87‍±‍0.21 vs. 3.65‍±‍0.22 J·kg‍1‍¹·m‍1‍¹; equivalent to 0.93 vs. 0.87 kcal·kg‍1‍¹·km‍1‍¹, *p* ₍FDR₎‍=‍0.01). EC increased progressively with higher VT2-relative intensities in both conditions. At 70–79% of VT2, EC was significantly higher during overground running compared with treadmill running (3.87‍±‍0.21 vs. 3.65‍±‍0.22 J·kg‍1‍¹·m‍1‍¹; p-FDR‍<‍0.01). At 90–100% of VT2, EC reached 4.60‍±‍0.27 J·kg‍1‍¹·m‍1‍¹ during overground running compared with 4.10‍±‍0.18 J·kg‍1‍¹·m‍1‍¹ during treadmill running (p-FDR‍<‍0.01). Across all domains, field running consistently demonstrated greater energetic demands, reflected by a higher intercept (3.6451‍±‍0.0736 J·kg‍1‍¹·m‍1‍¹) and a steeper EC-intensity slope (0.0354‍±‍0.0039 J·kg‍1‍¹·m‍1‍¹ per %VT2) compared with treadmill running (intercept: 3.5418‍±‍0.0704 J·kg‍1‍¹·m‍1‍¹; slope: 0.0188‍±‍0.0033 J·kg‍1‍¹·m‍1‍¹ per %VT2), as shown in Fig 5.

### Substrate utilization

Substrate oxidation rates across intensity domains are presented in Table 2. At running speeds below VT₁ (8–10 km·h‍1‍¹), fat oxidation (0.61‍±‍0.12 vs. 0.58‍±‍0.12 g·min‍1‍¹) and carbohydrate oxidation (1.74‍±‍0.35 vs. 1.90‍±‍0.35 g·min‍1‍¹) did not differ significantly between treadmill and overground conditions (p‍>‍0.10 for both). In contrast, between VT₁ and VT2, a progressive shift toward carbohydrate metabolism was observed in both environments. Overground running demonstrated significantly greater carbohydrate oxidation than treadmill running across this domain (3.20‍±‍0.80 vs. 2.75‍±‍0.60 g·min‍1‍¹; Δ‍=‍+0.45 g·min‍1‍¹, p-FDR‍=‍0.02, dz‍=‍0.65), with a corresponding trend toward lower fat oxidation during overground running (0.28‍±‍0.16 vs. 0.39‍±‍0.14 g·min‍1‍¹; Δ‍= −0.11 g·min‍1‍¹, p‍=‍0.07, dz‍=‍0.38), consistent with a progressive shift in substrate utilization toward carbohydrate as intensity approached VT2.

**Table 2:** Fat and carbohydrate oxidation rates during treadmill and overground running across ventilatory threshold-relative intensity domains.

Parameter	Treadmill (Mean‍±‍SD)	Overground (Mean‍±‍SD)	Δ [95% CI]	p-value	dz
Below VT₁ (8–10 km·h‍1‍¹)					
Fat oxidation (g·min‍1‍¹)	0.61‍±‍0.12	0.58‍±‍0.12	−0.03 [−0.12,‍+‍0.06]	>0.10	0.12
Carbohydrate oxidation (g·min‍1‍¹)	1.74‍±‍0.35	1.90‍±‍0.35	+0.16 [−0.16,‍+‍0.48]	>0.10	0.18
Between VT₁ and VT2 (11–13 km·h‍1‍¹)					
Fat oxidation (g·min‍1‍¹)	0.39‍±‍0.14	0.28‍±‍0.16	−0.11 [−0.23,‍+‍0.01]	0.07	0.38
Carbohydrate oxidation (g·min‍1‍¹)	2.75‍±‍0.60	3.20‍±‍0.80	+0.45 [+0.08,‍+‍0.82]	0.02*	0.65
Approaching VT2 (12–13 km·h‍1‍¹)					
Fat oxidation (g·min‍1‍¹)	0.36‍±‍0.14	0.15‍±‍0.12	−0.21 [−0.33, −0.09]	0.01*	0.72
Carbohydrate oxidation (g·min‍1‍¹)	3.03‍±‍0.58	3.70‍±‍0.65	+0.67 [+0.28,‍+‍1.06]	0.01*	0.75

Note. Values are mean‍±‍SD. Δ‍=‍mean difference (overground – treadmill) with 95% CI. p-values are FDR-adjusted. * p‍<‍0.05. Fat and carbohydrate oxidation rates calculated from simultaneous V̇O2 and V̇CO2 using non-protein stoichiometric equations (Frayn, 1983). Stages with RER‍>‍1.00 excluded. Below VT₁‍=‍stages at 8–10 km·h‍1‍¹; VT₁-VT2 domain‍=‍stages at 11–13 km·h‍1‍¹; peak stage‍=‍12 km·h‍1‍¹ stage specifically.

## Discussion

The primary aim of this study was to evaluate the energetic and metabolic consequences of applying the commonly used 1% treadmill grade correction when comparing laboratory and overground running in trained endurance athletes. The novelty of the present work lies in its integrated technical evaluation of the 1% treadmill grade correction using multiple energetic outcomes RE, O2-COT, and EC anchored to individually determined ventilatory thresholds, rather than arbitrarily defined submaximal speeds. In contrast to the meta-analysis by Miller et al. (2019), which focused primarily on time-normalized V̇O2 and did not account for the specific 1% grade correction applied, nor for substrate utilization when interpreting energetic cost, the present study extends the literature by quantifying whether meaningful energetic differences persist between overground and 1%-grade treadmill running when multiple energetic outcomes and substrate oxidation data are considered together. [14]. The key finding of the present study was that RE, O2-COT, and EC were all consistently higher during overground running than treadmill running at a 1% grade across the intensity domains examined, with divergence occurring primarily at intensities between 70% and 100% of VT2, where minimal differences were observed below this threshold. For RE, the overground slope was steeper (b‍=‍0.4189 vs. 0.3446 mL·kg‍1‍¹·min‍1‍¹ per %VT2), indicating that not only were absolute RE values consistently higher during overground running, but the rate of increase with intensity was also greater overground. This suggests that the mechanisms driving the overground RE elevation surface variability, postural demands, and aerodynamic resistance impose a progressively greater energetic overhead as intensity increases toward VT2, rather than contributing a fixed cost across all intensities. By contrast, the treadmill’s mechanical efficiency advantage appears to be better preserved as speed increases, reflected by the shallower RE-intensity slope. In the current cohort, VT2 occurred at approximately 13.1–13.5 km·h‍1‍¹, providing a clear physiological reference point. These findings indicate that the 1% correction does not fully replicate the energetic demands of overground running, particularly at higher submaximal intensities. Although Miller et al. (2019) reported broadly comparable V̇O2 responses between treadmill and overground running below ~16 km·h‍1‍¹, those conclusions were based on oxygen uptake per unit time and did not incorporate distance-normalized outcomes; our data suggest that when more sensitive metrics are used, the 1% correction proves insufficient, especially near VT2 [14]. These findings provide meaningful technical insight into the limitations of the 1% adjustment: despite being the most widely applied correction in endurance running research, the 1% grade does not fully replicate the energetic demands of overground running, particularly as intensity approaches VT2. Collectively, these results suggest that laboratory-based assessments using a 1% grade may systematically underestimate the energetic cost of overground running at race-relevant intensities, with practical implications for training prescription and performance evaluation. The consistently higher RE during overground running across all intensity domains suggests that the mechanical and environmental demands of track running impose a greater steady-state oxygen cost than treadmill running at a 1% grade, even when the correction specifically intended to equalise these demands is applied. It should be noted that the 1% treadmill grade correction is mechanistically imprecise. Mesquita et al. (2024) demonstrated that a 1% grade may substantially overestimate or underestimate the contribution of air drag depending on running speed, and therefore does not consistently reproduce the primary mechanical difference between laboratory and overground running [15]. Rather than attributing potential differences solely to the absence of air resistance, differences in energetic cost between conditions may reflect the combined influence of surface mechanics, locomotor variability, and aerodynamic demand that extend beyond what the 1% correction was designed to address.

The steeper RE-intensity slope observed during treadmill running (b‍=‍0.4189 vs. 0.3446 mL·kg‍1‍¹·min‍1‍¹ per %VT2) indicates that the relative increase in oxygen cost per unit increase in intensity was greater on the treadmill, likely reflecting the constrained and mechanically consistent nature of treadmill locomotion where small increases in belt speed translate directly to proportional increases in metabolic demand. In contrast, the shallower slope but higher absolute RE values during overground running suggest a baseline elevation in oxygen cost attributable to surface variability, postural adjustment demands, and aerodynamic resistance factors that persist across intensities rather than scaling linearly with speed. These findings are consistent with previous observations that overground locomotion imposes additional stabilization demands not present during treadmill running [33,34]. The oxygen cost of transport was consistently higher during overground running, with significant differences emerging at faster speeds (Fig 3). From 70–79% of VT2 onward, O2-COT increased by +7.1 mL·kg‍1‍¹·km‍1‍¹ (dz‍=‍0.59). These findings are consistent with previous work by di Prampero et al. (1986), who identified O2-COT as an integrative descriptor of locomotor oxygen cost and reported that while energy cost per unit distance was independent of treadmill speed under controlled laboratory conditions, accounting for air resistance during overground running meaningfully improved the accuracy of endurance performance prediction [33]. [34]. It is worth noting that O2-COT would theoretically remain relatively constant with increasing exercise intensity if V̇O2 and speed increased proportionally. However, the progressive increase in O2-COT observed in both conditions above VT₁ is consistent with the emergence of a V̇O2 slow component, a well-established loss of muscular efficiency above VT₁ (Poole & Jones, 2012) that causes V̇O2 to rise disproportionately relative to running speed [35]. Critically, this efficiency loss appears more pronounced during overground running, as reflected by the steeper O2-COT slope (0.7538 vs. 0.5855 mL·kg‍1‍¹·km‍1‍¹ per %VT2), suggesting that the additional mechanical and neuromuscular demands of overground running amplify the intensity-dependent efficiency loss beyond what is observed during treadmill running at a 1% grade. While O2-COT captures the oxygen cost per unit distance, EC extends this by integrating substrate-specific energetic yield, making it sensitive to both the volume of oxygen consumed and the metabolic fuel used. The consistently higher EC during overground running across all intensity domains, with the between-condition difference growing from 0.22 J·kg‍1‍¹·m‍1‍¹ at 70–79% of VT2 to 0.50 J·kg‍1‍¹·m‍1‍¹ at 90–100% of VT2, indicates that the energetic consequences of the 1% correction become progressively more apparent as intensity approaches VT2. The divergence between EC and V̇O2 responses, where EC differences exceeded V̇O2 differences between conditions, reflects the additional contribution of substrate shift: greater carbohydrate oxidation during overground running yields less energy per litre of O2 consumed than fat oxidation (~21.1 vs.‍~‍19.6 kJ·L‍1‍¹ O2 respectively), thereby amplifying the EC difference beyond what V̇O2 alone would predict. This makes EC a more sensitive discriminator of condition differences than RE or O2-COT, and supports its inclusion as a primary outcome in studies evaluating the energetic consequences of the 1% correction. Substrate utilization patterns revealed that below VT₁ (8–10 km·h‍1‍¹), fat and carbohydrate oxidation rates were comparable between treadmill and overground conditions (p‍>‍0.10 for both), consistent with the predominantly aerobic, fat-based metabolism characteristic of moderate-intensity running. As exercise intensity rose between VT₁ and VT2, carbohydrate oxidation became significantly greater during overground running compared with treadmill running (3.20‍±‍0.80 vs. 2.75‍±‍0.60 g·min‍1‍¹; Δ‍=‍+0.45 g·min‍1‍¹, p-FDR‍=‍0.02, dz‍=‍0.65), with a corresponding trend toward lower fat oxidation during overground running (0.28‍±‍0.16 vs. 0.39‍±‍0.14 g·min‍1‍¹; p‍=‍0.07) [36]. This shift is consistent with an earlier reliance on carbohydrate metabolism during overground running at matched speeds. The present findings demonstrate that carbohydrate oxidation was significantly greater during overground running between VT₁ and VT2 despite comparable V̇O2 values at lower intensities. Notably, this difference emerged progressively as intensity approached VT2, suggesting an intensity-dependent mechanism rather than a constant metabolic offset between conditions. These observations indicate that the additional mechanical and neuromuscular demands of overground running preferentially increase glycolytic flux at higher intensities, without a proportional elevation in whole-body oxygen uptake. We consider three mechanisms physiologically plausible, and explain the reasoning behind each. First, overground running on a track surface imposes greater stride-to-stride mechanical variability than treadmill running, requiring continuous small-amplitude postural corrections via stabilizing musculature. These stabilizing muscles are predominantly composed of fast-twitch and type IIa fibres, which have a higher glycolytic capacity and a lower fat-oxidation rate than slow-twitch fibres [37]. Their repeated recruitment near VT2 would therefore increase carbohydrate oxidation rates and elevate the respiratory exchange ratio without a proportional rise in whole-body V̇O2, precisely the pattern observed here. Second, aerodynamic drag during overground running is not fully offset by the 1% grade correction, particularly at speeds above 12 km·h‍1‍¹ [15]. The additional mechanical work required to overcome residual air resistance is performed at a metabolic intensity that favours carbohydrate as the primary substrate, given that fat oxidation capacity is progressively limited as exercise approaches VT2 [38]. Third, treadmill belt motion passively assists the leg recovery phase, reducing the muscular work required during swing; this assistance is absent during overground running, increasing total neuromuscular demand per stride and shifting the metabolic burden toward glycolytic pathways at higher submaximal intensities. We acknowledge that these mechanisms remain inferential in the absence of direct biomechanical and muscle fibre recruitment data. However, we consider them physiologically coherent and consistent with the observed pattern of elevated RER and elevated carbohydrate oxidation, with no large differences in V̇O2 between conditions. Future studies incorporating electromyography, force plate measurements, and fibre-type specific metabolic assessments would be required to confirm the relative contribution of each mechanism. Our findings extend prior work demonstrating that substrate utilization during incremental exercise is sensitive to both exercise intensity and locomotor context. Specifically, previous studies have shown that running surface and mechanical variability can influence the metabolic substrate mix independently of V̇O2 [39,40], and that the intensity at which the crossover from fat to carbohydrate predominance occurs is modifiable by environmental and biomechanical factors [41]. Building on these observations, our findings indicate that the substrate shift toward carbohydrate predominance occurs at lower absolute speeds during overground running than during treadmill running at a 1% grade, suggesting that the additional mechanical and neuromuscular demands not replicated by this conventional correction are sufficient to lower the intensity at which glycolytic pathways become dominant.

### Study limitations

Several limitations of the present study should be acknowledged. First, at exercise intensities above VT₁, bicarbonate buffering of hydrogen ions generates non-metabolic CO2 that elevates measured RER above its true metabolic value. Consequently, RER-based stoichiometric equations may overestimate carbohydrate oxidation rates and underestimate fat oxidation rates at intensities between VT₁ and VT2 [38]. Although stages with RER‍>‍1.00 were excluded from substrate analyses, some degree of RER inflation below this threshold cannot be entirely excluded. Second, the V̇O2 slow component above VT₁ may delay attainment of true metabolic steady state beyond three minutes [35]. While the steady-state verification criteria applied V̇O2 slope‍≤‍100 mL·min‍1‍¹·min‍1‍¹ and CV‍≤‍5% during the final 60 seconds were designed to minimise this effect, complete steady-state attainment at intensities approaching VT2 cannot be guaranteed. These two limitations should be considered when interpreting the substrate utilization findings. Third, while the study provides a detailed metabolic and ventilatory comparison between overground and treadmill running, direct biomechanical and neuromuscular measurements were not included. Variables such as ground reaction forces, stride-to-stride variability, muscle activation patterns, and mechanical work distribution were not assessed. As a result, the mechanistic explanations proposed for the observed differences in energetic cost, including altered stabilization demands, surface-related mechanics, and aerodynamic resistance, remain inferential and should be regarded as physiologically plausible hypotheses rather than confirmed mechanisms. Furthermore, as only male athletes were recruited in the present study, generalizability of these findings to female athletes is limited. Future research should examine whether similar differences in RE, O2-COT, EC, and substrate utilization between treadmill and overground running are observed in female endurance athletes, particularly given the potential influence of menstrual cycle phase on ventilatory and metabolic responses. The order of treadmill and field testing was not randomized, with all participants completing the laboratory assessment prior to the field-based session. Although a minimum of 48–72 hours was allowed between sessions to minimize residual fatigue, potential order effects cannot be fully excluded. Future studies should employ a counterbalanced or randomized crossover design to control for familiarization and carry-over effects.

## Conclusion

In conclusion, this study provides technical insight into the limitations of the commonly used 1% treadmill grade correction. Although maximal oxygen uptake was comparable between conditions, both running economy and oxygen cost of transport were higher during overground running. These differences became more pronounced at intensities approaching and exceeding the second ventilatory threshold. These findings indicate that overground running is associated with a higher energetic cost per unit distance. While the present study did not directly assess environmental or locomotor mechanisms, the observed differences are consistent with the interpretation that the 1% correction does not fully replicate the energetic demands of overground running. By integrating oxygen uptake with substrate-specific energy equivalents, the present study provides additional insight into the energetic and metabolic consequences of the 1% correction and highlights the possibility that laboratory-based assessments using a 1% grade may underestimate energetic cost at race-relevant intensities.
